# Living in Western Australia induces some physiological adaptations of seasonal acclimatisation in the surgical burns team

**DOI:** 10.1080/23328940.2023.2281210

**Published:** 2023-11-07

**Authors:** Zehra Palejwala, Karen E. Wallman, Grant J. Landers, Prashan Anbalagan, Fiona M. Wood, Shane K. Maloney

**Affiliations:** a School of Human Sciences (Sports Science Exercise and Health), The University of Western Australia, Crawley, WA, Australia; b Burn service of Western Australia, WA Department of Health, Nedlands, WA, Australia; c Burn Injury Research Unit, University of Western Australia, and Burn service of WA South Metropolitan Health Service, Perth, WA, Australia

**Keywords:** Seasonal acclimatization, burn surgery, adaptations, heat tolerance, heat stress, core temperature, heart rate

## Abstract

Seasonal acclimatization is known to result in adaptations that can improve heat tolerance. Staff who operate on burn injuries are exposed to thermally stressful conditions and seasonal acclimatization may improve their thermoeffector responses during surgery. Therefore, the aim of this study was to assess the physiological and perceptual responses of staff who operate on burn injuries during summer and winter, to determine whether they become acclimatized to the heated operating theater. Eight staff members had physiological and perceptual responses compared during burn surgeries conducted in thermoneutral (CON: 24.1 ± 1.2°C, 45 ± 7% relative humidity [RH]) and heated (HOT: 31.3 ± 1.6°C, 44 ± 7% RH) operating theaters, in summer and winter. Physiological parameters that were assessed included core temperature, heart rate, total sweat loss, sweat rate, and urinary specific gravity. Perceptual responses included ratings of thermal sensation and comfort. In summer, CON compared to winter CON, baseline (85 ± 15 bpm VS 94 ± 18 bpm), mean (84 ± 16 bpm VS 93 ± 18 bpm), and peak HR (94 ± 17 bpm VS 105 ± 19 bpm) were lower (*p* < 0.05), whereas core temperature was not different between seasons in either condition (*p* > 0.05). In HOT, ratings of discomfort were higher in summer (15 ± 3) than winter (13 ± 3; *p* > 0.05), but ratings of thermal sensation and sweat rate were similar between seasons (*p* > 0.05). The surgical team in burns in Western Australia can obtain some of the physiological adaptations that result from seasonal acclimatization, but not all. That is most likely due to a lower than required amount of outdoor heat exposure in summer, to induce all physiological and perceptual adaptations.

## Introduction

During major surgery to repair extensive burn injuries that involve ~ 20% of a patient’s total body surface area (TBSA), it is common practice to heat the operating theater (OT) to temperatures between 30°C and 40°C [1]. The elevated OT temperature can mitigate inadvertent intraoperative hypothermia (core temperature [T_CORE_] <36°C; [2]), a phenomenon that occurs often in the anesthetized patient during surgery [3]. While the elevated OT temperature improves patient outcomes [3], it also creates issues for heat balance in surgery staff. Ambient heat combined with physical activity, the wearing of personal protective equipment (PPE), and no fluid intake, can compromise thermoregulation in surgery staff, resulting in an increase in T_CORE_, sweating, and dehydration [4–7]. An increase in T_CORE_ (≥38.5°C is classified as hyperthermia; [8]) and dehydration can result in thermal discomfort [9], heat illness [10], cognitive impairment [11], and detriment to physical performance [12], all of which are concerning in an OT where a patient’s life is at stake and optimal performance is required.

To the best of our knowledge, only one study has explored the physiological responses of surgery staff during real-time burn surgery in the OT. That study compared a control condition (24°C, 45% RH) to a hot condition (30.8°C, 39% RH; [13]). Compared to the control condition, the hot surgery resulted in a higher T_CORE_, heart rate (HR), sweat loss, and more dehydration, all of which can contribute to heat illness, thermal discomfort, and impaired performance, in combination or alone. Similar results were also noted during a simulated burn surgery [14]. For that reason, it is important to find ways to mitigate the effects of heat stress in burn surgery staff.

Frequent exposure to hot environments can result in adaptations that improve tolerance to hot ambient conditions [15]. The adaptation is known as acclimatization/acclimation, which occurs as a result of regular exposure to ambient or artificial ambient heat but can also occur in response to physical activity, without exposure to ambient heat [16]. Seasonal acclimatization occurs due to seasonal changes in ambient conditions [15], while acclimation occurs in response to artificially imposed thermal stress [17]. The physiological adaptations that are associated with acclimatization include a lower resting T_CORE_, a lower resting HR, lower sweating thresholds, higher maximum sweat rate, an expanded plasma volume, and improved fluid and electrolyte balance. Perceptual adaptations include a lowered rating of perceived exertion and thermal sensation, and an improved rating of thermal comfort when exposed to heat [16,18,19]. While researchers have explored the influence that acclimation can have on occupational thermal strain [20,21], the potential for seasonal acclimatization has not been explored in depth in an occupational context, specifically one in which heat stress is a common occurrence. A lower resting T_CORE_ and HR have been reported in summer compared to winter, in individuals living in in Korea [22] and Japan [23], respectively, where individuals experience differences in ambient temperatures between seasons. It is possible that staff working in heated OTs could benefit from acclimatization over the summer months in Western Australia where outdoor temperatures can exceed 40°C.

Therefore, the aim of this study was to assess the physiological and perceptual responses of staff during burn surgery in the summer and winter months, to explore whether they acclimatize in the summer when outdoor temperatures are high. It was hypothesized that staff working in hot and thermoneutral OTs in the summer months would exhibit a lower baseline and mean T_CORE_ and HR, a higher total sweat loss and sweat rate, a lower rating of thermal sensation, and improved comfort levels than during the winter months, due to the effects of seasonal acclimatization.

## Materials and methods

### Participants

Surgical and hospital staff (*n* = 8) from a burns department were tested in both control (CON: 24°C) and hot (HOT: 31°C) OT conditions in summer and winter (Table 1). A power analysis was not conducted but a convenience sample approach was used for this study due to inconsistency in the occurrence and staffing during heated, burns theaters. All staff gave written, informed consent for participation in this study and patients gave either written or verbal consent (written consent could not be provided by all patients because of the nature and location of the burn injury). Verbal consent was witnessed by a member of the surgical burns team who then signed and dated the consent form, attesting that the requirements for informed consent were satisfied. Ethical approval was granted by the Human Research Ethics Committee of the University of Western Australia (2020/ET000239) and the Human Research Ethics Committee of the South Metropolitan Health Service (PRN RGS0000004250) in Perth, Western Australia.

**Table 1.** Participant numbers and demographic information for summer and winter, in CON and HOT surgery conditions (mean ± SD).

		Males	Females	Surgeons	Scrub nurses	Age (years)	Height (cm)	Mass (kg)
Summer and Winter	CON	2	5	4	3	50 ± 8	165 ± 6	80 ± 17
	HOT	2	6	5	3	49 ± 8	166 ± 6	82 ± 18

#### Experimental design

In the CON condition, seven participants were assessed over a total of nine surgeries in summer and 13 surgeries in winter. In the HOT condition, eight participants were assessed over a total of six surgeries in summer and five surgeries in winter (Table 2). Testing in the summer months occurred over January-March, when the daily average, maximum, ambient temperature was 32.1 ± 2.4°C and testing in the winter months occurred over June–October, when the daily average, maximum, ambient temperature was 19.9 ± 1.5°C. All surgeries commenced between 8:30 am and 9:30 am and the staff wore the same standard surgical clothing and PPE (scrub gown, gloves, scrub hat, surgical mask) in each trial. Surgeries in which the TBSA of the burn injury of patients was >20% were classified as severe [24].

**Table 2.** Number of testing sessions per participant (ID) in CON and HOT surgery conditions, in Summer and Winter.

	ID	1	2	3	4	5	6	7	8
CON	Summer	○	○○	○	○○○	○	○○	○○○	
	Winter	○○	○○○○	○○	○○○○	○○	○○○○	○○○○	
HOT	Summer	•••	••	••	•	•	•	•	••
	Winter	••	•	•	•••	•	•••	••	••

Note: The dates for CON and HOT surgeries in summer are as follows. CON: 12/01, 13/01, 25/01, 02/02, 09/02, 17/02, 24/02, 09/03, 19/03, 25/03 HOT: 18/01, 19/01, 08/02, 03/03, 17/03, 30/03

#### Familiarization session

Each participant attended a familiarization session approximately one week prior to their first assessed surgery, where they were made familiar with the perceptual questionnaires and had their height and body-mass measured. The female staff were asked to provide information on their menstrual cycle and contraception that was used to determine which phase of the menstrual cycle they were in during each observation. The participants then completed the International Physical Activity Questionnaire (IPAQ; [25]) to estimate total physical activity in metabolic equivalent (MET) and in minutes/week, as well as the type and intensity of physical activity completed per week, to determine the potential for acclimatization/acclimation. None of the staff had traveled to a warmer climate in the months prior to testing. The participants were familiarized with the testing equipment including HR monitors (Polar RS400, Finland), weighing scale (SOEHNLE, Style sense comfort 100, Digital personal scale and Anko Glass Electronic Personal Scale, Model no. EB9373), pedometers (ActiGraph wGT3X+, Florida, USA), and the refractometer (for determining urine specific gravity [U_SG_]: ATAGO MASTER-URC/Na, Tokyo, Japan). The values obtained for U_SG_ were classified as “well hydrated” (<1.010), “minimal dehydration” (1.010–1.020), “significant dehydration” (1.021–1.030), or “serious dehydration” (>1.030; [26]). The participants were also provided with a T_CORE_ capsule (CorTemp, HQ Inc., Palmetto, USA; [27]) along with an instruction sheet, and were asked to ingest the capsule 4–8 h prior to the start of surgery. That time frame is recommended to allow the pill to pass from the stomach to the small intestine for a more stable T_CORE_ reading [27].

#### Protocol

Upon arrival at the hospital, the staff provided a urine sample to determine U_SG_. In private, nude body mass was measured to the nearest 0.1 kg using a digital platform scale, after which the staff were fitted with a HR monitor and pedometer. Steps were recorded via the pedometer as an indicator of activity level during surgery, as higher levels of activity can increase T_CORE_ and HR [28]. In the OT, prior to surgery, the perceptual questionnaires were completed. Staff then exited the OT (~2–3 min) to scrub before surgery began, and then they fulfilled their usual roles within the OT. An initial (baseline) T_CORE_ and HR measurement was taken as soon as surgery commenced and again at 15-min intervals throughout surgery. Once surgery began, the staff remained in the OT and did not consume any food/fluids until after the final measures were recorded at the conclusion of the surgery. The perceptual questionnaires were completed again at the completion of surgery, followed by the measurement of nude body mass to determine total sweat loss (pre nude body mass – post nude body mass), and the collection of a final urine sample.

#### Perceptual questionnaires

The participants rated their thermal sensation (TS) and thermal comfort (TC) using a 20-point scale from “very cold” to “very hot,” and “very comfortable” to “very uncomfortable,” respectively [29]. A score of 10 indicated neutral thermal sensation and comfort.

#### Statistical analysis

The data were analyzed using R Studio (Version 1.4.1717). A linear mixed model (obtained using the *lmer* function), specifying participant ID as a random-effect variable, was used to assess all dependent variables, across all time points (specific to U_SG_, TS and TC) and both seasons, through the *anova* test function. Linear mixed-model regressions were used to assess the influence of environmental conditions in the OT on outcome variables. Significance was accepted at *p* ≤ .05. All results are expressed as mean ± SD. Due to our sample size, Hedges’s *g* effect sizes (ES) with ± 95% confidence intervals (CI) were also calculated. Only large (*g*  ≥ 0.76) and moderate (*g* = 0.38–0.75) ES are reported [30,31].

## Results

Environmental conditions in the OT during the CON trials were 24.1 ± 1.3°C, 45.7 ± 7.4% RH, 1.37 ± 0.25 kPa (water vapor pressure [WVP]) in summer, and 24.0 ± 1.1°C, 44.5 ± 6.4% RH, 1.33 ± 0.20 kPa in winter. There was no seasonal difference in OT temperature (*p* = .953), relative humidity (*p* = .338), or WVP (*p* = .307). The OT conditions during the HOT trials were 31.7 ± 1.4°C, 47.4 ± 4.1% RH, 2.22 ± 0.27 kPa in summer, and 30.8 ± 1.6°C, 38.6 ± 7.1% RH, 1.73 ± 0.39 kPa in winter. The OT temperature (*p* = .011), humidity (*p* < .001), and WVP (*p* < .001) were higher in summer than winter. Surgery duration was not different between seasons in either condition (CON; winter: 141 ± 50 min, summer:143 ± 42 min; *p* = .907 and HOT: winter: 158 ± 51 min, summer: 171 ± 53 min; *p* = .512). In the CON condition, the participants registered more steps in winter (460 ± 249) than summer (217 ± 141; *p* = .004). In the HOT condition, the participants registered more steps in summer (447 ± 276) than winter (271 ± 137; *p* = .045). Burn injury TBSA of patients in the CON condition was 5 ± 6% in winter and 6 ± 9% in summer, while in the HOT condition it was 20 ± 7% in winter and 33 ± 23% in summer.

Of the six females tested, three were post-menopausal and two were using an intrauterine device, which meant that their menstrual cycle was un-identifiable. One female was tested a total of four times; twice in summer and twice in winter. In both seasons one testing session was in the luteal phase and one was in the follicular phase.

### Physical activity and heat exposure

The IPAQ estimated that most participants undertook high intensity physical activity (*n* = 7) each week, and one undertook moderate intensity physical activity (*n* = 1) each week (mean = 8,232 ± 4,248 MET and range = 2,070–15,162 MET per week). Within the testing period (February 2021-February 2022), there were a total of 12 heated surgeries performed by the surgical staff, with the mean duration of exposure per surgery being 166 ± 53 min, equating to a mean heat-exposure time of 40 min per week over the year (details provided in Table 3).

**Table 3.** Information on participant physical activity and heat exposure in the OT.

	IPAQ RESULTS							OPERATING THEATRE	
	RECREATION Indoor/Outdoor not specified			OUTDOOR GARDEN		WEEKLY ESTIMATION (including indoor, job related activity and transport)		HEAT EXPOSURE	
Participant	Walk	Moderate	Vigorous	Housework (moderate)	Housework (vigorous)	METs	Category	Self-reported estimate per week (hours)	Quarterly exposure (hours)
1	7	3.5	3.5	3	0	15162	High	4	7
2	0	0	0	0	0	2070	Moderate	2	3.75
4	4	0	3	0.75	4	8613	High	3	4.5
5	2	0	0	0.5	0	5052	High	12	5
6	2.5	1	2.25	0	5	9258	High	8	1.75
7	2.25	0	0	0	0	8805	High	6	6.75
8	2	0	0	3	0	4665	High	8	4
9	2.25	9	1	0	0	12228	High	5	6.5

Numbers (for recreation, outdoor garden, and self-reported estimate of OT heat exposure) denote hours per week. Weekly estimation of physical activity is given in METS with the equivalent intensity of physical activity participants were estimated to partake in. *Quarterly exposure denotes actual hours each participant worked in a hot OT over three-months.

### Core temperature

**Figure 1.**
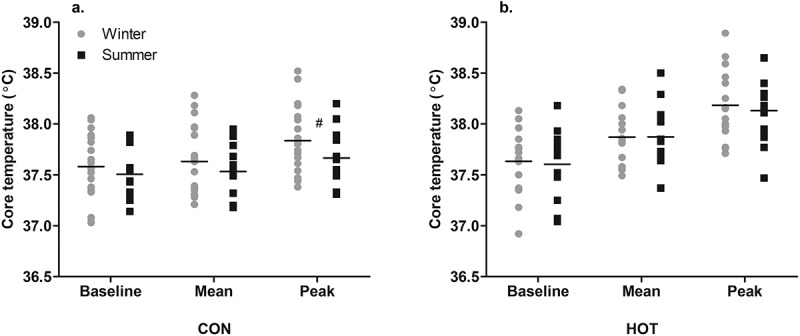
Seasonal T_CORE_ responses in CON (a) and HOT (b) conditions.

In the CON condition, while the ANOVA found no effect of season on the baseline T_CORE_ (*p* = .799), mean T_CORE_ (*p* = .671), peak T_CORE_ (*p* = .189), or change in T_CORE_ (peak T_CORE_ minus baseline T_CORE;_ *p* = .188), the ESwas moderate, suggesting that peak T_CORE_ was lower in summer (37.67 ± 0.30°C) than winter (37.83 ± 0.32°C; *g* = 0.47 [−0.27, 1.21]) and change in T_CORE_ was smaller in summer (0.16 ± 0.21°C) than winter (0.25 ± 0.17°C; *g* = 0.45 [−0.29, 1.19]; Figure 1)

# Moderate effect size between seasons in specified condition (*g* = 0.47).

Individual and mean data shown

In the HOT condition, there was no effect of season on the baseline T_CORE_ (*p* = .778), mean T_CORE_ (*p* = .946), or peak T_CORE_ (*p* = .185), indicating similar values in the heat, between seasons. The mean change in T_CORE_ was 0.55 ± 0.3°C in winter and 0.53 ± 0.33°C in summer, with no difference between seasons (*p* = .113; Figure 1). Accounting for differences in OT conditions in HOT, in which temperature and WVP were higher in summer than winter, neither OT temperature nor WVP influenced baseline T_CORE_ or the change in T_CORE_ (*p* > .05).

### Heart rate

**Figure 2.**
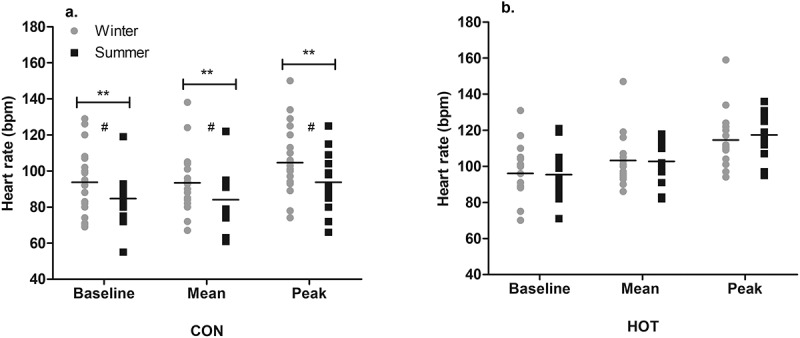
Seasonal HR responses in CON (a) and HOT (b) conditions.

In the CON condition, the baseline HR was lower in summer (85 ± 15 bpm) than winter (94 ± 18 bpm; *p* = .008; *g* = 0.49 [−0.21, 1.18]), the mean HR during surgery was lower in summer (84 ± 16 bpm) than winter (93 ± 18 bpm; *p* = .004; *g* = 0.48 [−0.21, 1.17]), and peak HR during surgery was lower in summer (94 ± 17 bpm) than winter (105 ± 19 bpm; *p* = .004; *g* = 0.55 [−0.14, 1.25]). The mean change in HR (peak minus baseline) was 9 ± 11 bpm in summer and 11 ± 8 bpm in winter, with no significant difference between the two (*p* = .486; Figure 2).

**Main effect (season): significant difference between seasons in specified condition and variable (*p* < .01);

# Moderate effect size between seasons in specified condition (*g* = 0.48-0.55). Individual and mean data shown

In the HOT condition, there was no effect of season on the baseline HR (*p* = .681), mean HR (*p* = .948), or peak HR (*p* = .729), indicating that HR was unchanged between seasons. The mean change in HR during surgery was 22 ± 12 bpm in summer and 18 ± 13 bpm in winter, with no significant difference between the two (*p* = .903; Figure 2). Neither OT temperature nor WVP influenced baseline HR or the change in HR (*p* > .05).

### Total sweat loss

**Figure 3.**
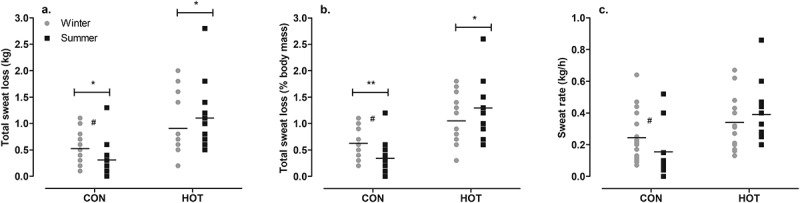
Total sweat loss in kg (a), total sweat loss as a percentage of body mass (b), and sweat rate (c) in CON and HOT surgeries, in summer and winter.

In the CON condition, there was a significant difference between seasons in the total sweat loss during surgery (*p* = .011; *g* = 0.55 [−0.17, 1.26]), with more sweat lost in winter (0.5 ± 0.3 kg) than in summer (0.3 ± 0.4 kg). When the total sweat loss is expressed as a percentage of total body mass there was a larger % BM loss in winter (0.6 ± 0.3%) than summer (0.3 ± 0.3%; *p* = .007; *g* = 0.92 [0.19, 1.66]) in the CON condition. When the total sweat loss is expressed as a rate (sweat rate; kg/h), while the ANOVA found no effect of season (*p* = .074), the ES was moderate, suggesting that sweat rate was higher in winter (0.24 ± 0.15 kg/h) than in summer (0.16 ± 0.18 kg/h; *g* = 0.46 [−0.25, 1.17]; Figure 3).

*Main effect (season): significant difference between seasons in specified condition and variable (*p* < .05);

**Main effect (season) significant difference between seasons in specified condition and variable (*p* < .01);

# Moderate effect size between seasons in specified condition (*g* = 0.46-0.92). Individual and mean data shown.

In the HOT condition, there was a significant effect of season on the total sweat loss during surgery (*p* = .016), with higher values in summer (1.1 ± 0.6 kg) than winter (0.9 ± 0.6 kg). When the total sweat loss is expressed as a percentage of total body mass a similar outcome emerged; there was a significant difference between seasons (*p* = .011), with values being higher in summer (1.3 ± 0.5%) than winter (1.1 ± 0.5%). When the total sweat loss is expressed as a rate (kg/h), there was no difference between seasons in the HOT condition (*p* = .316; Figure 3). Neither OT temperature nor WVP influenced the sweat loss or sweat rate (*p* > .05).

### Urinary specific gravity

**Figure 4.**
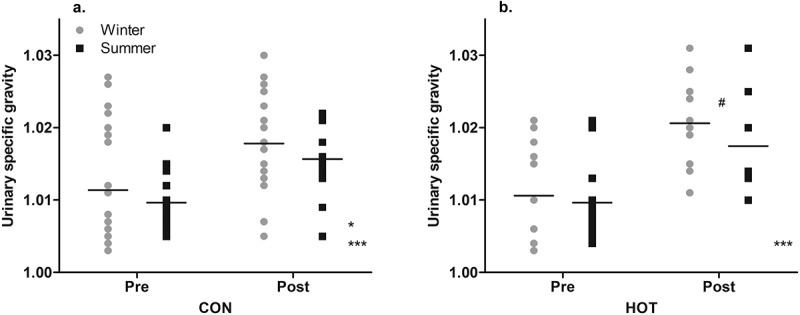
“Pre” and “post” U_SG_ values in CON (a) and HOT (b) surgeries, in summer and winter.

In the CON condition, the U_SG_ scores post-surgery (1.017 ± 0.006) were higher than pre-surgery (1.011 ± 0.008; *p* < .001; *g* = 0.85 [0.35, 1.34]). Overall, in the CON condition, there was a significant effect of season (*p* = .046), with participants being more dehydrated in winter (1.015 ± 0.008) than summer (1.013 ± 0.006). There was no interaction between season and time on scores for U_SG_ (*p* = .698; Figure 4).

*Main effect (season): significant difference between seasons in specified condition (*p* < .05);

***Main effect (time): significant difference between scores pre- and post-surgery in specified condition and (*p* < .001);

#Moderate effect size between seasons at specified time point and in specified condition (*g* = 0.57).

Individual and mean data shown

In the HOT condition, the U_SG_ scores post-surgery (1.019 ± 0.007) were higher than pre-surgery (1.010 ± 0.006; *p* < .001; *g* = 1.28 [0.68, 1.88]). While the ANOVA found no effect of season on U_SG_ scores in the HOT condition (*p* = .880), and no interaction between season and time (*p* = .518), the ES was moderate, suggesting lower values of U_SG_ post-surgery in the summer (1.017 ± 0.007) than in winter (1.021 ± 0.006; *g* = 0.57 [−0.25, 1.39]; Figure 4). Temperature influenced scores for U_SG_, whereby as temperature increased, scores for U_SG_ decreased (*t*(46) = −2.499, *p* = .016)).

### Thermal sensation

**Figure 5.**
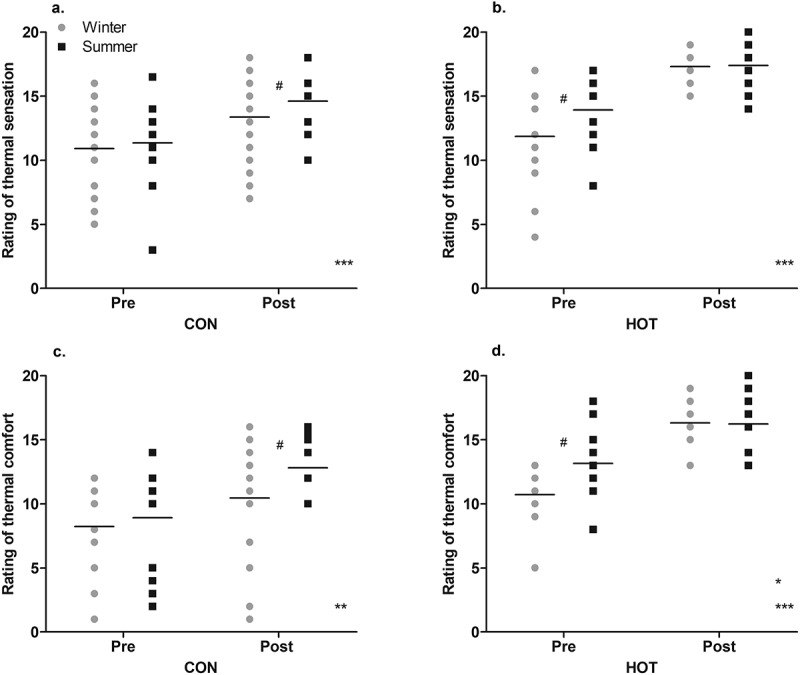
“Pre” and “post” perceptual responses in summer and winter; thermal sensation CON (a), thermal sensation HOT (b), thermal comfort CON (c), and thermal comfort HOT (d).

In the CON condition while the ANOVA found no effect of season on ratings of thermal sensation (*p* = .232), the ES was moderate, indicating that post-surgery, the ratings of thermal sensation were higher (the staff felt hotter) in summer (15 ± 3) than in winter (13 ± 3; *g* = 0.62 [−0.09, 1.32]). There was a significant effect of time (*p* < .001), with the ratings post-surgery (14 ± 3) being higher than pre-surgery (11 ± 3; i.e. the staff felt hotter post-surgery). There was no interaction between season and time (*p* = .570; Figure 5).

*Main effect (season): significant difference between scores in summer and winter in specified condition (*p* < .05); ** Main effect (time): significant difference between scores pre- and post-surgery in specified condition (*p* < .01);

***Main effect (time): significant difference between scores pre- and post-surgery in specified condition and (*p* < .001);

#Moderate to large effect size between seasons at specified time point and in specified condition (*g* = 0.52–0.73).

Individual and mean data shown.

In the HOT condition while the ANOVA found no effect of season on ratings of thermal sensation (*p* = .262), the ES was moderate, indicating that pre-surgery, the ratings of thermal sensation were higher (the staff felt hotter) in summer (14 ± 3) than in winter (12 ± 4; *g* = 0.52 [−0.24, 1.27]). There was a significant effect of time (*p* < .001), with higher scores post-surgery (17 ± 2) than pre-surgery (13 ± 3). There was no interaction between season and time (*p* = .109; Figure 5). Neither OT temperature nor WVP in the HOT condition influenced the ratings of thermal sensation (*p* > 0.05).

### Thermal comfort

In the CON condition while the ANOVA found no effect of season on ratings of thermal comfort (*p* = .059), the ES was moderate, indicating that post-surgery, the ratings of thermal comfort were higher (the staff felt more uncomfortable) in summer (13 ± 2) than winter (10 ± 5; *g* = 0.66 [−0.04, 1.37]). There was a significant effect of time (*p* = .001), with the ratings post-surgery (11 ± 4) being higher than pre-surgery (8 ± 4; i.e. the staff felt more uncomfortable post-surgery). There was no interaction between season and time (*p* = .357; Figure 5).

In the HOT condition, the ratings of thermal comfort were higher in summer (15 ± 3) than winter (13 ± 3; *p* = .040). There was a significant effect of time (*p* < .001), with scores being higher post-surgery (16 ± 2) than pre-surgery (12 ± 3). There was an interaction between time and season (*p* = .031), with post hoc analysis indicating a smaller difference between ratings of thermal comfort pre- and post-surgery in summer (*p* = .002) than winter (*p* < .001) and higher ratings pre-surgery in summer (13 ± 3) than winter (11 ± 2; *p* = .022, *g* = 0.73 [−0.03, 1.50]; Figure 5). The difference in WVP between summer and winter of the OT in the HOT condition, influenced ratings of thermal comfort whereby as WVP increased, ratings of thermal comfort decreased (*t*(50) = −2.100, *p* = .041)).

## Discussion

To our knowledge, this is the first study to assess for the potential for seasonal acclimatization within an occupational setting, specifically one in which heat stress is a common occurrence. The results from this study suggest that living in Western Australia, where significant ambient temperature differences occur between summer and winter, can induce some physiological adaptations of seasonal acclimatization, in surgical staff who operate on burn injuries.

The baseline, mean, and peak HR were lower in summer than winter, in the CON condition, which is consistent with research that reported a lower resting HR in summer than winter (by 5 bpm) in participants, in a thermoneutral climate chamber (25°C, 50% RH; [32]). Keatisuwan and colleagues (1996) similarly reported that the resting HR of participants following seasonal acclimatization was 9 bpm lower in summer than in winter [23], the same as the reduction in baseline HR that was noted in our study. The resting HR values in summer in the aforementioned studies were 74 bpm [32] and 71 bpm [23], whereas the mean, baseline HR value in summer in our study was 85 bpm. That could mean that the staff were simply fitter in summer, as regular exercise and physical activity can cause a reduction in resting HR [33]. However, information regarding physical activity levels was collected in one season only, so it is difficult to make a comparison of seasonal fitness levels. While the measurements taken in this study were baseline, they were likely not true resting measurements that are typically taken in a relaxed, seated position, which might provide a suggestion as to why the baseline values in our study were higher than those previously documented.

While seasonal acclimatization is a likely explanation for our finding that HR was lower in summer, other parameters that are normally associated with acclimatization were not different between seasons. Acclimatized individuals have a lower resting T_CORE_ [15], but we saw no seasonal difference in T_CORE_. The time course of adaptations to heat is faster for HR than for T_CORE_ [34] and so it is possible that our subjects were partly, but not yet fully acclimatized. Differences in resting T_CORE_ of 0.05°C [35] to 0.14°C [22] between summer and winter have been documented and there was a 0.08°C difference in our baseline T_CORE_ between seasons. It is possible that the lack of significance between summer and winter in baseline T_CORE_ was a result of a small sample size (CON; *n* = 7), or simply an indication that the staff were not seasonally acclimatized in terms of T_CORE_.

After seasonal acclimatization, one would typically see a smaller increase in HR, and a smaller change in T_CORE_ during heat exposure or physical activity, as those are changes that are associated with acclimatization [15,36]. Most studies that assess whether acclimatization occurrs in individuals, do so by measuring outcome variables during a heat response test that involves exposure to a standard, hot environment, using a fixed workload and duration [15]. The heated OT in the present study could be viewed as another form of a standard heat exposure, and we did not see seasonal differences in the HR or T_CORE_ responses to that exposure. An explanation for the lack of seasonal differences in HR and T_CORE_ responses during the heated surgeries could be the effect of heightened psychological stress during the more complex surgeries in summer [37,38], compared to winter. Anticipation of a complex surgery could result in autonomic stimulation of the sympathetic nervous system [39] and stress-induced thermogenesis [40], and mask any potential effect of acclimatization. Surgery complexity, indicated by TBSA of the patients’ injuries, averaged 33% in summer and 20% in winter. Patients with an injured site equating to > 20% TBSA are classified as severe cases [41], and those cases often equate to a more complex surgery as they result in the release of inflammatory mediators at the site of injury [24].

Studies that have reported the adaptations of seasonal acclimatization have generally looked at participants who had ≥10 hours of outdoor heat exposure per day [22,42]. The participants in the present study reported undertaking an average of 6 hours of recreational physical activity per week, but the questionnaire did not differentiate between indoor and outdoor physical activity. They also reported an average of 2 hours of physical activity outdoors in the garden per week. So, all we can say for certain is that our participants spent at least 2 hours outside per week. That value is lower than the necessary “outdoor heat exposure” to induce physiological adaptations to hot conditions. In general, hospital staff spend most of their work time indoors in an air-conditioned hospital, and continuous exposure to an air-conditioned environment is likely to hinder acclimatization to the local weather [43].

In the CON condition, the total sweat loss as an absolute value, and relative to body mass, were higher in winter, while sweat rate was the same. The difference of 0.2 kg (absolute value) and 0.3% (relative to body mass) may be due to the higher step count/activity that the staff accumulated during CON surgery in the winter, however, it equated to a difference of only 243 steps over ~2 hours, approximately one extra step per minute. In the HOT condition, the total sweat loss as an absolute value, and relative to body mass, was higher in summer (1.3%) than winter (1.1%) and others have reported similar differences between seasons. For example, Hori (1997) reported that sweat loss increased from 1.04% body mass in winter to 1.37% body mass in summer during 2 hours of passive heat exposure [44], and Lei and colleagues (2021) reported that sweat loss increased from 0.9% body mass in winter to 1.1.% body mass in summer after 1 hour of cycling [32], with both studies documenting that change as an adaptation of seasonal acclimatization. In this study, the higher total sweat loss relative to body mass in the summer may be a result of seasonal acclimatization or could be attributed to the higher registered steps of participants in summer. After seasonal acclimatization, a higher sweat rate is expected during heat exposure [15], however sweat rate in the HOT condition was similar between seasons. That could be due to a small sample size or may be an indication that the staff were not seasonally acclimatized in terms of sweating responses.

The staff became dehydrated (higher scores for U_SG_) during surgery in both conditions, and both seasons, which was to be expected as none of the staff consumed fluids during surgery that lasted a few hours. In the CON condition, the staff became more dehydrated in winter than summer, which was most likely due to the higher mass loss in winter. The scores for U_SG_ were similar between seasons in the HOT condition, but post-surgery, the slightly lower scores in summer indicate better hydration, despite mass loss being higher in summer. Before surgery, although not significant, it appears in the HOT condition most subjects had a U_SG_ value <1.010 in summer, while in winter they tended to be >1.010, which provides a possible explanation as to why the staff were slightly less dehydrated post-surgery in summer.

Pre-surgery, in the HOT condition, the staff felt slightly hotter and more uncomfortable (moderate ES) in summer than winter, which is most likely due to the higher ambient temperature and humidity conditions in the OT in summer. The ratings of thermal sensation were similar between seasons but levels of discomfort were higher in summer than winter. We expected the staff would be more comfortable in summer]as the perception of thermal comfort in the heat tracks seasonal variations in ambient temperature and thus higher temperatures are tolerated better by individuals in the summer [45]. However, it is difficult to compare ratings of thermal comfort between seasons as, in this study, the higher WVP in summer in the HOT condition, although not having an impact on T_CORE_, HR or sweating responses, had an impact on ratings of thermal comfort.

### Limitations and future directions

The main limitation of this study was the small sample size, potentially leading to an underpowered analysis. Additionally, the baseline T_CORE_ and HR measurements were not taken immediately when the staff entered the OT, but approximately 10 minutes after, just prior to the commencement of surgery. If during those 10 minutes the physiology of the staff had already changed in response to the OT conditions, then it was not a true resting measurement. The average temperature and WVP in the OT was 31.7 ± 1.4°C, 2.22 ± 0.27 kPa in summer, and 30.8 ± 1.6°C, 1.73 ± 0.39 kPa in winter in the HOT condition (i.e. higher in summer than winter). The environmental conditions were something we could not control and therefore our data represent the real world environment in burn surgery. Skin temperature, which is an important indicator of cardiovascular stress and a crucial feedforward mechanism in thermoregulation, was not assessed. Lastly, the difference in patient TBSA in HOT, between summer and winter, confounds our seasonal comparison of the physiological responses in that condition. It would be beneficial to assess physiological responses, including additional parameters like plasma volume and sweat composition, of surgery staff during heated surgeries of the same complexity to accurately assess for seasonal acclimatization. Because physiological measures can affect performance, future research should build on this study and assess mental and physical performance in the more complex surgeries (>20% TBSA), where physiological responses may be heightened as a result of the increased OT temperature combined with psychological stress.

## Conclusion

This study showed that living in Western Australia, where ambient temperatures change between seasons, can induce some physiological adaptations of seasonal acclimatization. To elicit the full state of heat acclimatization, the members of the surgical burns team may be required to increase their outdoor heat exposure in summer months. Obtaining the full state of seasonal acclimatization may enhance the burns team’s tolerance to heat, thereby minimizing heat strain when repairing extensive burn injuries in a hot OT.

## Data Availability

The data that supports the findings of this study are available from the corresponding author [ZP] upon reasonable request.

## Abbreviations

HR: Heart rate
IPAQ: International physical activity questionnaire
MET: Metabolic equivalent
OT: Operating theatre
PPE: Personal protective equipment
RH: Relative humidity
TBSA: Total body surface area
TC: Thermal comfort
T_core_: Core temperature
TS: Thermal sensation
U_SG_: Urinary specific gravity
WVP: Water vapour pressure
